# The Effect of Co-administration of 4-Methylcatechol and Progesterone on Sciatic Nerve Function and Neurohistological Alterations in Streptozotocin-Induced Diabetic Neuropathy in Rats

**Published:** 2011-04-21

**Authors:** Hamidreza Sameni, Marzieh Panahi

**Affiliations:** 1. Department of Anatomical Sciences, Faculty of Medicine, Semnan University of Medical Sciences, Semnan, Iran; 2. Department of Anatomical Sciences, Faculty of Medicine, Ahwaz Jondishapour University of Medical Sciences, Ahwaz, Iran

**Keywords:** Diabetic Neuropathy, Sciatic Nerve, Progesterone, 4-Methylcatechol, Rat

## Abstract

**Objective:**

Diabetic neuropathy is the most common complication of diabetes mellitus affecting the nervous system. In this study, we investigated the *in vivo* effects of combined administration of 4-methylcatechol (4-MC) and progesterone (P) as a potential therapeutic tool for sciatic nerve function improvement and its role in histomorphological alterations in diabetic neuropathy in rats.

**Materials and Methods:**

Male adult rats were divided into 3 groups: sham operated control (CO), untreated diabetic (DM) and diabetic treated with progesterone and 4-methylcatechol (DMP4MC) groups. Diabetes was induced by a single dose injection of 55 mg/ kg streptozotocin (STZ). Four weeks after the STZ administration, the DMP4MC group was treated with P and 4-MC for 6 weeks. Then, following anesthesia, the animals' sciatic nerves were removed and processed for light and transmission electron microscopy (TEM) as well as histological evaluation.

**Results:**

Diabetic rats showed a statistically significant reduction in motor nerve conduction velocity (MNCV), nerve blood flow (NBF), mean myelinated fiber (MF) diameters and myelin sheath thickness of the sciatic nerve after 10 weeks. In the sciatic nerve of the untreated diabetic group, endoneurial edema and increased number of myelinated fibers with myelin abnormalities such as infolding into the axoplasm, irregularity of fibers and alteration in myelin compaction were also observed. Treatment of diabetic rats with a combination of P and 4-MC significantly increased MNCV and NBF and prevented endoneurial edema and all myelin abnormalities.

**Conclusion:**

Our findings indicated that co-administration of P and 4-MC may prevent sciatic nerve dysfunction and histomorphological alterations in experimental diabetic neuropathy.

## Introduction

Diabetic peripheral neuropathy is the presence of symptoms and signs of peripheral nerve dysfunction in people with diabetes after exclusion of other causes. It occurs in more than 50-60% of diabetic patients and is the most frequently encountered neuropathy in developed countries with wide-ranging effects on its sufferers’ quality of life (1).

This disorder involves a spectrum of functional and structural changes in peripheral nerves such as decrease in motor nerve conduction velocity (MNCV), decrease in nerve blood flow (NBF), reduction of 'Na^+^/K^+^-ATPase'activity and loss of myelinated fibers which are the hallmarks of diabetic neuropathy (2). Earlier studies have demonstrated a relationship between structural nerve lesions and diminished nerve conduction velocity (NCV) in diabetic animals (3).

Current treatment of diabetic neuropathy relies on the control of glycemic and oxidative stresses as well as neural and vascular risk factors (4, 5). Recently, it has been reported that steroid hormones (neuroactive steroids) such as progesterone exert a broad spectrum of neuroprotective effects both in the central and peripheral nervous systems and can counteract the peripheral nerve degeneration occurring in experimental physical trauma, aging and in hereditary demyelization disease (6-9).

Neuroactive steroids may control proliferation of Schwann cells and the biosynthesis of their products such as myelin membranes, myelin proteins including glycoprotein zero(P0) and peripheral myelin protein 22 (PMP22) as well as transcription factors involved in the myelination process (4, 10, 11). Previous studies have demonstrated that progesterone (P) biosynthesis is up-regulated in the spinal cord and peripheral nerves of rats with STZ-induced diabetes (9).

Additionally, progesterone and its derivatives are also able to stimulate, both *in vivo* (e.g. in the rat sciatic nerve) and *in vitro* (e.g. in cultures of rat Schwann cells), the synthesis of two important peripheral nerve myelin proteins (12-15).

Recent studies have indicated that 4-methylcatechol (4-MC), as a catecholamine derivative and a nerve growth factor (NGF) degradation suppressor or NGF secretion stimulator, may be a potential therapeutic tool for the treatment of certain neurological disorders (16-18). There is a correlation between a reduced plasma NGF content and decreased sciatic nerve MNCV; furthermore, the administration of 4-MC preventeds a drop in the sciatic nerve MNCV and plasma NGF content of experimental diabetic neuropathic rats (17).

Previous studies have shown that in the pathogenesis of diabetic peripheral neuropathy, two very important pathways including nerve growth factor pathway deficiency and oxidative stress (production of free radicals and reactive oxygen and nitrogen species) have an important role (1, 4). Thus, the objective of the presented study is combined administration of 4-methylcatechol (as a stimulator of endogenous nerve growth factor synthesis) and progesterone (as a neuroactive steroid with antioxidant and neuroprotective properties) to determine whether these two substances can inhibit possible pathways in the pathogenesis of diabetic neuropathy in order to reduce complications and neurological problems caused by diabetes.

Therefore, in the presented study, the simultaneous effects of progesterone and 4-methylcatechol on functional parameters (NCV and NBF) and histomorphology of the sciatic nerve was examined in STZ-induced diabetic neuropathy in rats.

## Materials and Methods

### Animals

Thirty male adult Sprague- Dawley rats (200-220 g) were obtained from the laboratory animal center of Ahwaz Jondishapur University of Medical Sciences (AJUMS). They were maintained under constant conditions of light and darkness (12 hours light-dark cycles), controlled temperature (20 ± 2℃) and humidity (60-65%) in plastic cages. All animals were acclimatized for a minimum period of two weeks prior to the beginning of the study. Experimental procedures were approved by the ethics committee of AJUMS, Ahwaz, Iran.

### Experimental design and drug treatment

Animals were randomly divided into 3 groups (10 rats per group): a nondiabetic control group (CO), an untreated diabetes mellitus group (DM) and a progesterone and 4-methylcatechol treated diabetic group (DMP4MC). Diabetes in rats was induced by a single-dose intrapritoneal injection of freshly prepared 55 mg/kg streptozotocin (STZ) from Sigma-Aldrich, USA in a 0.09 M citrate buffer (pH=4.8) (19-21). Hyperglycemia was confirmed 48 hours after the STZ injection by measuring tailvein blood glucose levels using a blood glucose monitoring system (EasyGluco, Infopia Co. Ltd., Korea). Only animals with mean plasma glucose levels above 300 mg/dl were accepted as being diabetic (22-24). For drug therapy purposes, diabetic and control animals were age-matched. After confirmation of diabetes, all diabetic animals were maintained in the laboratory for 4 weeks to allow stabilization of their neuropathic process (25, 26). Four weeks after diabetic induction in the DMP4- MC group, they were treated with a combination of P (8 mg/kg, IP; Sigma-Aldrich, USA) dissolved in 200 µl sesame oil and 4-MC (10 µg/kg, IP; Sigma- Aldrich, USA) in PBS once every two days for 6 weeks. Animals in CO and DM groups received vehicle alone (8, 16, 17). At the end of experiment, all rats were sacrificed under anesthesia and their sciatic nerves were removed for morphological analysis.

### Motor nerve conduction velocity measurement

The animals were anesthetized with 50/20 mg/ kg ketamine/xylazine IP injections to prevent discomfort and then their right sciatic nerve MNCV was measured. During the study, the animals’ body temperatures were maintained at 37℃ using a warming pad to ease anesthesia stress. For MNCV measurement, sciatic-tibial stimulation was induced proximally at the sciatic notch level and distally at the knee level using bipolar platinum needle electrodes (0.5 mm diameter, 20 mm length; the stimulation period was 10 ms, frequency was 2-2000 Hz, 5-10 V, single stimulus) (27, 28). The nerve studies lasted less than 30 minutes per rat, and the electrodes were disinfected with 70% alcohol between animals to maintain a pathogen- free status. Recording needle electrodes connected to a bio-potential coupler were placed on the rats’ paws to detect motor response. The motor response was captured using PowerLab/4SP with Dual Bio Amp (ADInstruments, Australia). The recording was a typical biphasic response with an initial M-wave, which is a direct motor response due to stimulation of the motor fibers of the gastrocnemious muscle. The sciatic-tibial MNCV was calculated using two points of stimulation along the nerve and measuring the resultant latency. Latency was measured from initial onset to maximum negative peak. MNCV was calculated using the following formula: MNCV = distance between sciatic and tibial nerve stimulation points/sciatic M-wave latency - tibial M-wave latency (21, 29).

### Sciatic nerve blood flow measurement

NBF was measured using a laser Doppler flowmetry (LDF) system (MOOR Instruments, UK). Each animal was anaesthetized with a 50/20 mg/ kg ketamine/xylazine IP injection. The left flank sciatic nerve was then exposed and a laser probe was placed just above the nerve. The exposed nerve was covered with normal saline to avoid tissue dehydration during the study; body temperatures of the animals were also maintained at 37℃ using homoeothermic blanket systems and the sciatic nerve temperatures were monitored using digital thermometers. Rats were stabilized for 10-15 minutes, then a continuous NBF recording over a 10-minutes period was performed as described by Sayyed et al. (30). The blood flow was reported as arbitrary perfusion units.

### Morphological assessment

The sciatic nerve specimens between the sciatic notch and the knee were fixed in situ under ketamine/ xylazine anesthesia with 4% glutaraldehyde in a 0.1 M phosphate buffer solution (PBS; pH=7.4) for 20 minutes. Then, the nerves were rapidly removed, cut into 1-2 mm long segments and fixed in 2.5% glutaraldehyde in phosphate buffered saline (PBS) for 24 hours. Tissue samples were then washed in PBS, post fixed for 2 hours in 1% buffered osmium tetroxide and dehydrated in graded concentrations of acetone and embedded in epoxy resin (TAAB Laboratories, UK). Transverse semi-thin sections (0.75 µm) were stained with 1% toluidine blue and observed under a light microscope. Morphometric analysis was performed using a computerized image analysis system (Motic China Group Co., Ltd., China). In each section, myelinated fiber (MF) diameters and myelin sheath thicknesses were calculated. At least 200 MF diameters were measured in each animal's sciatic nerve. For ultrastructural study, ultrathin transverse sections (70-80 nm) were mounted on grids, stained with uranyl acetate and lead citrate and examined using a Philips EM300 transmission electron microscope equipped with a digital camera.

### Statistical analysis

The quantitative data have been analyzed by using the SPSS software. Data from experiments with more than two independent variables have been analyzed using the one-way analysis of variance (ANOVA) followed by the Tukey-Kramer posthoc tests. All data were expressed as the mean ± SEM and the differences were considered to be significant when p<0.05.

## Results

As shown in table 1, 10 weeks after STZ administration, diabetic rats in both DM and DMP4- MC had hyperglycemia; they also showed slight weight gain, although significantly less than rats in the CO group. Body weight of 10^th^ week diabetic rats was significantly (p<0.05) lower than that of nondiabetic control rats. Combined treatment with P+4-MC, did not significantly affect the blood glucose levels and body weights in diabetic rats.

**Table 1:** Body weight and blood glucose levels of CO, DM and DMP4MC groups


Animal	Body weight (g)		Blood glucose (mg/dl)
Groups	Before STZ injection	End of experiment	Before sacrifice

CO (n=10)	255.1 ± 3.8	384.9 ± 5.9	121 ± 24.185
DM (n=10)	245.7 ± 2.8	270.7 ± 3.8*	544 ± 53.508***
DMP4MC (n=10)	231.9 ± 2.7	277.1 ± 5.4*	496 ± 66.384***


Data are mean ± SEM, n is the number of animals studied in each group. *p<0.05 vs. control, ***p<0.001 vs. control.

### Electrophysiology

**Fig 1:**
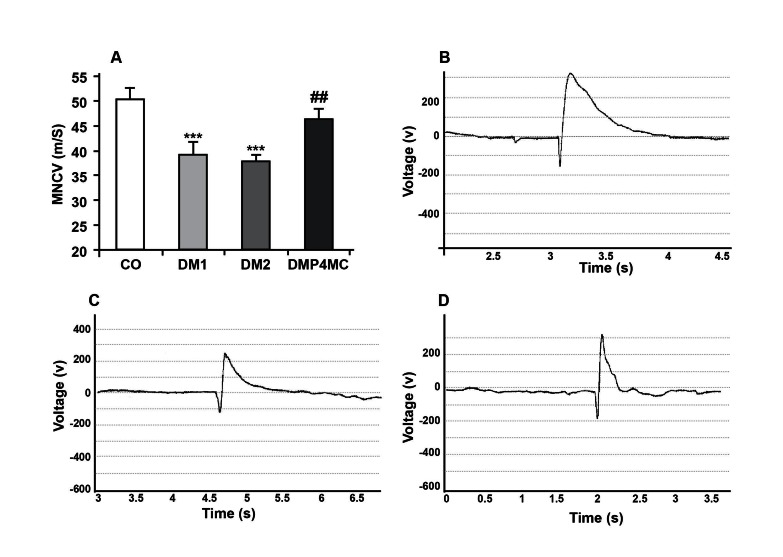
A. MNCV in the sciatic nerves of control (CO), untreated diabetic in 4 and 10 weeks (DM1 and DM2 respectively) after STZ injection and P+4MC-treated diabetic (DMP4MC) rats. MNCV in DM1 and DM2 groups were significantly slower than CO and DMP4MC. Three samples of sciatic nerve motor responses recorded from, B. the control, C. diabetic and D. P+4MC-treated diabetic groups. Data are shown as mean ± SEM, n = 10, *** p<0.001 vs. CO, ## p<0.01 vs. DM1 and DM2.

Before the P+4MC treatment (at the end of 4th week), MNCVs were significantly reduced (23%) in diabetic groups compared with the control group (p<0.001). The diabetic rats treated with P and 4-MC for six week showed a significant improvement (69%) in their MNCVs as compared with the not treated diabetic group (p<0.01) (Fig 1A). Fig 1 B-D show samples of sciatic nerve motor responses recorded in (B) control group, (C) diabetic group and (D) P+4MC-treated diabetic group.

### Sciatic nerve blood flow

**Fig 2:**
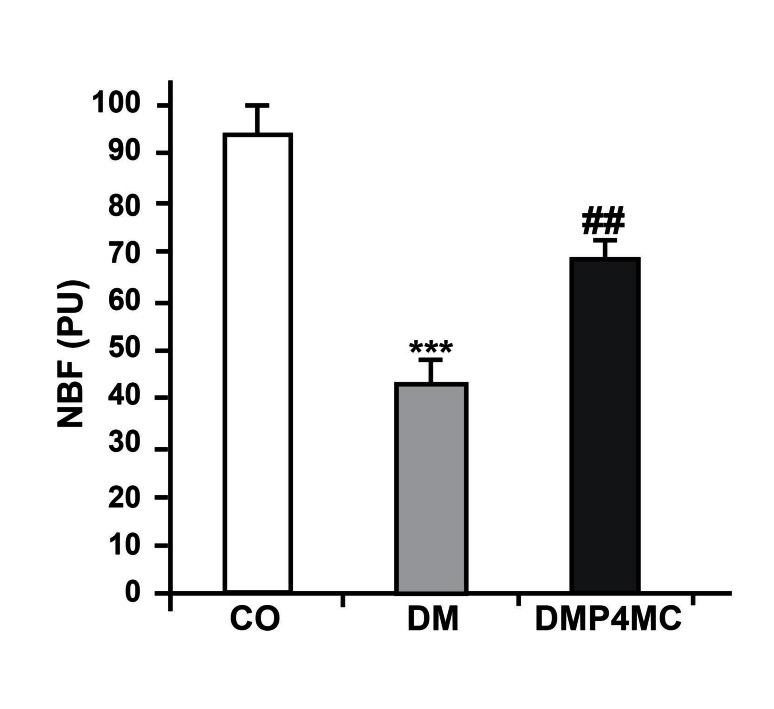
NBF in the sciatic nerves of control (CO), diabetic (DM) and P+4MC-treated diabetic (DMP4MC) rats. Data are shown as mean ± SEM, n = 10, *** p<0.001 vs. CO, ## p<0.01 vs. DM.

Composite nerve blood flow in untreated diabetic rats was significantly reduced (45%) compared with the control rats. A six-week combined treatment with P and 4-MC showed significant improvement (28%) in NBF of diabetic rats (Fig 2).

### Light Microscopy

**Fig 3:**
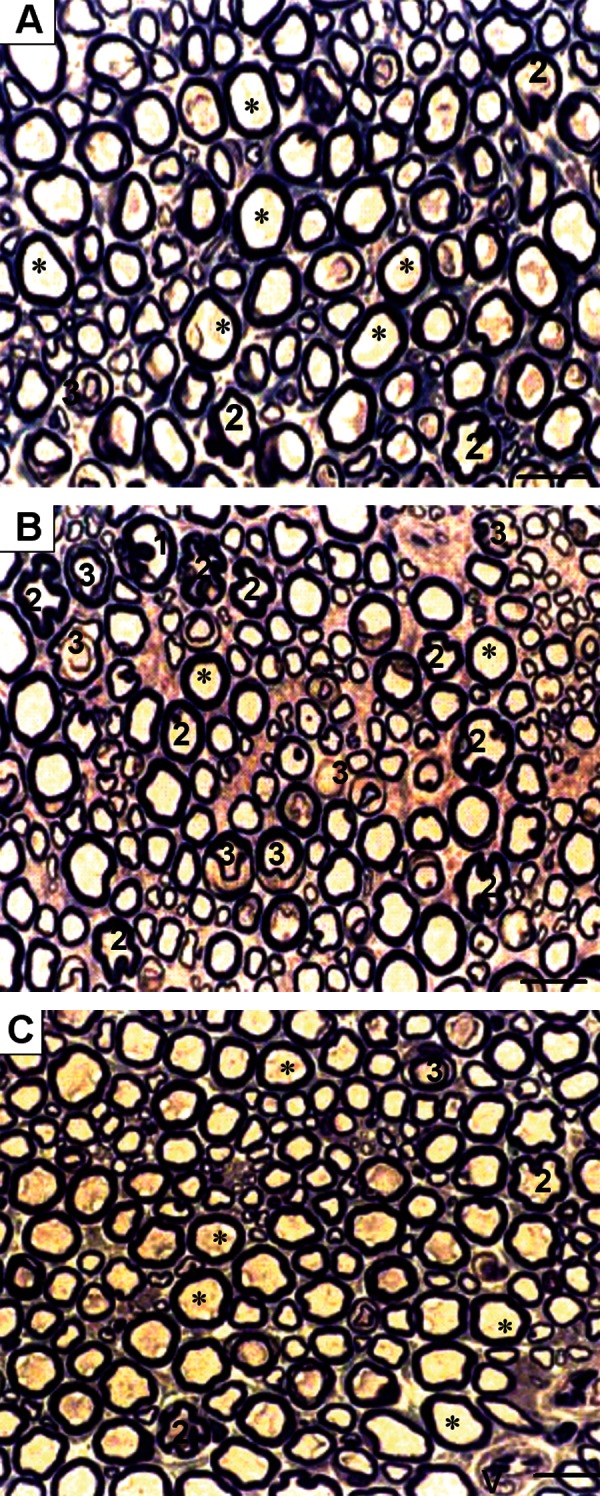
Light micrographs of toluidine blue stained transverse semi-thin section of the sciatic nerve at ×400 magnification, scale bar: 20 µm, V: vessel. (A) In the control group, myelinated nerve fibers are in normal morphology and structure (*). (B) In the untreated diabetic group, nerves revealed certain abnormalities, including: (1) degeneration, (2) myelin abnormalities including irregular fiber shapes, myelin infoldings and outfoldings and (3) alteration in myelin compaction. (C) In the P+4MC-treated diabetic group, the proportion of axons with myelin abnormality was significantly reduced. Also the number of small myelinated nerve fibers was increased in the diabetic group as compared with the other two groups.

**Fig 4:**
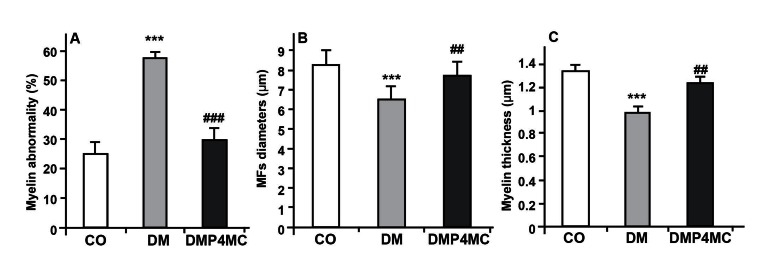
(A) Proportion of fibers with myelin abnormality, (B) myelinated fiber (MF) diameters and (C) Myelin thickness of the sciatic nerves of control, DM and DMP4MC groups. Data are mean ± SEM. *** significant differences vs. control and DMP4MC groups (p<0.001), ## significant differences vs. DM group (p<0.01), ### significant differences vs. DM group (p<0.001).

Light microscopic observations of semithin sections of the sciatic nerve from untreateddiabetic animals revealed some abnormalities including: endoneurial edema with dissociation of nerve fibers, degeneration, irregularity, infolding (myelin invaginations in the axoplasm), outfolding (myelin evaginations in the Schwann cell cytoplasm), derangement in myelin compaction and unclear boundary in myelin sheaths compared with control and DMP4MC groups (Fig 3A, B, C). These findings were in agreement with previous studies (3, 6, 31, 32). The most frequent of these abnormalities was the existence of myelin infoldings in the axoplasm. The proportion of fibers with myelin abnormalities (including infoldings, outfoldings and irregular shapes) in the sciatic nerve of the diabetic group was significantly reduced after six weeks of treatment with P and 4-MC (p<0.001, Fig 4A). The mean MF count and myelin thickness (µm) were significantly decreased in untreated diabetic rats compared with nondiabetic rats; furthermore, these changes were less severe in diabetic rats treated with P and 4-MC (p<0.001, Fig 4B, C).

**Fig 5:**
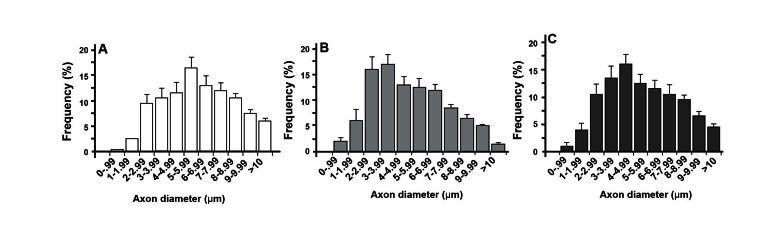
Comparison of axon diameter distribution of (A) the control group, (B) the diabetic group and (C) the DMP4MC group. Axon diameter distribution of the diabetic group and DMP4MC group shifted toward a smaller diameter compared with the control group. But this change was milder in the DMP4MC group compared to the diabetic group. Synergic effects of P and 4MC significantly restored the number of large MFs to their near normal values.

The distribution of myelinated axon diameter measurements in the three groups is shown in figure 4. The number of small myelinated nerve fibers was increased in the diabetic group and in all three groups a decrease was observed in the number of large myelinated fibers (Fig 5A, B). In addition, a shift in the peak of axon diameters to smaller sizes was also observed in the DMP4MC group, but this change was less manifested than in the diabetic group (Fig 5C).

### Electron microscopy

**Fig 6:**
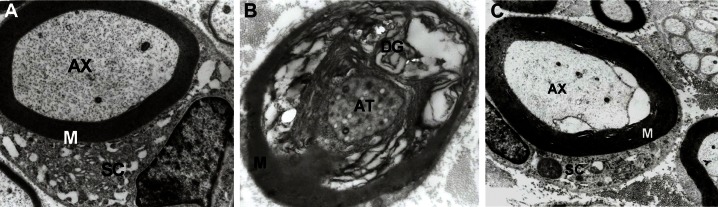
Electron micrographs of sciatic nerve transverse sections of (B) diabetic myelin degenerative (DG) and axonal atrophy (AT) seen in abnormally myelinated nerve fibers compared to control (A) and DMP4- MC (C) groups. AX: Axon; SC: Schwann cell; M: Myelin; (×14500 magnification).

The results of our ultrastructural study show extensive axonal degeneration and axonal atrophy in abnormal myelinated nerve fibers in the diabetic group compared with control and DMP4MC groups (Fig 6A, B, C) (3, 6, 29, 30).

In the control group, a compactly arranged myelinated axon and Schwann cells was observed; however, in the diabetic group, the myelinated nerve fibers were irregular and loosely arranged and myelin debris were enclosed within myelin sheaths. After 6 weeks, these changes in the sciatic nerves of diabetic animals were partially hindered by the treatment with P and 4-MC (Fig 6).

## Discussion

In this study, we have evaluated the protective effects of P and 4-MC on the development of diabetes- related neuropathy and measured the functional and structural parameters of the sciatic nerve specifically. The presented results indicate that rats with diabetic neuropathy showed a significant decrease in their MNCV and NBF, whereas nerve degeneration and irregularity (infolding and outfolding of myelin), abnormality in myelin compaction and the numberof small myelinated fibers increased in comparison with the control rats, representing a decline in nerve function and structure. However, simultaneous treatment of diabetic rats with P and 4-MC significantly improved these parameters.

Clinical and experimental studies have confirmed the presence of significant alterations in morphological and functional parameters in the peripheral nerves of humans and animals affected by diabetes. Neuropathy induced by diabetes is the result of relevant alterations of the nervous microvascular (vasa nervorum) that cause: axonal atrophy and degeneration, segmental demyelination, and hypertrophy and proliferation of Schwann cells (33). On the other hand, the previous study indicated that development of diabetic neuropathy in STZ-induced diabetic rats was evident from reduction in MNCV and NBF.

In the present study, we observed 18.5% and 23% deficit in MNCV at 4 and 10 weeks post diabetes induction in comparison with nondiabetic rats respectively. These results are consistent with previous studies reporting similar reductions of MNCV in STZ-induced diabetic rats (8, 29, 30, 34). We observed a 45% decrease in sciatic NBF after 10 weeks in untreated diabetic rats. This deficit in composite NBF was significantly improved thru simultaneous treatment of diabetic rats with P and 4-MC.

According to previous reports, Na^+^/K^+^-ATPase activity, nerve blood flow reduction, accumulation of polyol pathway metabolites in the sciatic nerve and reductions in axonal diameters may be major causes for decreased MNCV in STZ-diabetic models (3, 35).

Several reports indicated that peripheral diabetic neuropathy is a hypoxic neuropathy. Increased free radicals and oxidative stress under hyperglycemic conditions causes vascular impairment leading to decreased NBF and consequently endoneurial hypoxia and impaired neural function which may cause slowing of MNCV (25, 36, 37). Also NBF deficits resulting in ischemic-hypoxia may reduce MNCV directly through nerve energy depletion, or indirectly through oxidative stress and other secondary metabolic derangements in conducting nerve fibers (27).

In the presented study combined treatment with P (8 mg/kg) and 4-MC (10 µg/kg) produced a significant improvement in the motor nerve conduction deficits of up to 69% in the diabetic rats in comparison with untreated diabetic rats. This improvement could be due to significantly improved NBF, preserved fiber and axon diameters, improved Na^+^/ K^+^-ATPase activity and the prevented histological nerve damage.

Another important effect of treatments with P and 4-MC was a reduction in the frequency of axon and myelin abnormalities including axonal degeneration, myelin infolding and outfolding, derangement of myelin compaction and fiber irregularity. This is in accordance with other reports regarding diabetic neuropathy such as axonal atrophy and degeneration, segmental demyelination, splitting and ballooning of the myelin sheath and Schwann cell abnormalities (29, 38, 39). Therefore, our findings indicate that P and 4-MC are able to reduce morphological changes associated with diabetic neuropathy of the sciatic nerve. Morphological myelin abnormalities may be the consequence of alterations in myelin compaction due to diabetes-associated changes in myelin proteins.

In earlier studies, as well as in our study, the occurrence of myelin abnormalities in normal peripheral nerves such as myelin infoldings and irregularly shaped nerve fibers probably reflects a basal level of nerve fiber damage due to stretch or other mechanical loads (6, 40, 41).

Recent observations have indicated that increased endoneurial edema and histological damages in diabetic rats could result from altered sodium cell gradients related to impared Na^+^/K^+^-ATPase activity (35). However, in diabetic models, the decrease in Na^+^/K^+^-ATPase activity could be due to metabolic abnormality and histological damage. It seems that the simultaneous treatment of diabetic rats with P and 4-MC prevents histopathological nerve damage and endonurial edema; this is possibly due to improved Na^+^/K^+^-ATPase activity (35).

These findings, in accordance with previous studies, indicate that treatment with STZ increases morphological alterations in myelinated fibers of the sciatic nerve. The most abundant myelin abnormalities observed in our study were axoplasm myelin infolding and irregularly shaped neurofibers. Also, the our quantitative study of myelinated nerve fibers clearly showed that myelin sheaths were unaffected by diabetes. Myelin infolding is associated with alterations in myelin proteins such as P0, PMP22 and myelinassociated glycoprotein (MAG). All of these myelin proteins are important for the maintenance of multilamellar structure and myelin compaction of the peripheral nervous system. The frequency of myelin infolding is increased with aging and different peripheral neuropathies including peripheral diabetic neuropathy (6, 40). Recent reports have shown that P induces the expression of myelin proteins in Schwann cell cultures and in sciatic nerves of adult rats, whereas 4-MC stimulates the synthesis and secretion of NGF in astroglial cells (8, 11, 18). Therefore, it is conceivable that combined treatments with P and 4-MC induce changes in the expression of myelin proteins, and that the synthesis of NGF may be the cause of reduction in myelin abnormalities as well as increased remyelination of myelinated fibers in animals treated with these compounds.

In agreement with earlier findings, our presented data indicate that a significant mean MF diameter and myelin thickness occurred in STZ-induced diabetic rats (6). These may be results of the massive increase of small size MFs observed in diabetic sciatic nerves. Combined treatment of diabetic rats with P and 4-MC is able to counteract the decreases in mean MF diameter and myelin thickness of the sciatic nerve.

Previous studies suggest that the nerve fibers density is an important factor indicating the progress of restoration in sciatic nerve. Furthermore, neuroactive steroids (e.g. progesterone) and catecholamines (e.g. 4-MC) are capable of facilitating the nerve repair process (29). Probably, P and 4-MC through the induction of protein expression in peripheral myelin sheaths, increase Schwann cell activity; they also stimulate nerve growth factor synthesis and expression of extracellular matrix proteins which can increase myelinated axon diameters and the total number of nerve fibers per unit area thru which they provide structural repair in nerve fibers changes of diabetic animals (10, 11, 18).

## Conclusion

Our findings demonstrate that combined administration of P and 4-MC provides beneficial effects on long term diabetic neuropathy via improving sciatic nerve function and and counteracting histomorphological alterations in its fibers. Although further studies should determine the functional implications and mechanisms of these protective effects of P and 4-MC, our findings suggest that the use of these compounds may be considered as a potential therapeutic approach to maintaining peripheral nerve integrity in diabetic peripheral neuropathy.
